# ZEB1 Promotes Epithelial-Mesenchymal Transition of Endometrial Epithelial Cells and Plays a Critical Role in Embryo Implantation in Mice

**DOI:** 10.1007/s43032-024-01646-0

**Published:** 2024-09-01

**Authors:** Zhong Xu, Huan-Huan Yang, Hou-Zhi Chen, Bi-Zhen Huang, Ming Yang, Zhen-Hua Liao, Bi-Qing Xiao, Hong-Qin Chen, Jing Ran

**Affiliations:** 1 The First Affiliated Hospital of Xiamen University, School of Medicine, Xiamen University, 55 Zhenhai Road, Xiamen, 361003 PR China; 2 School of Clinical Medicine, Fujian Medical University, Fuzhou, 350001 Fujian PR China; 3 Duke Kunshan University, Duke University, Durham, NC 27708 USA

**Keywords:** Zinc finger E-box binding homeobox 1, Embryo implantation, Epithelial–mesenchymal transition, E-cadherin, Vimentin, Endometrial receptivity

## Abstract

Zinc finger E-box binding homeobox 1 (ZEB1) promotes epithelial-mesenchymal transition (EMT) in carcinogenesis, but its role in embryo implantation has not yet been well studied. In the present study we evaluated the hypothesis that ZEB1-induced EMT is essential for embryo implantation in vivo. Endometrial epithelium from female Kunming mice (non-pregnant, and pregnant from day 2.5 to 6.5) were collected for assessment of mRNA/protein expression of ZEB1, and EMT markers E-cadherin and vimentin, by employment of real-time quantitative reverse transcription PCR, Western blot, and immunohistochemical staining. To test if knockdown of ZEB1 affects embryo implantation in vivo, mice received intrauterine injection of shZEB1 before the number of embryos implanted was counted. The results showed that, ZEB1 was highly expressed at both mRNA and protein levels in the mouse endometrium on day 4.5 of pregnancy, paralleled with down-regulated E-cadherin and up-regulated vimentin expression (*P* < 0.05). Intrauterine injection of shZEB1 markedly suppressed embryo implantation in mice (*P* < 0.01). Conclusively, the present work demonstrated that ZEB1 is essential for embryo implantation under in vivo condition, and is possibly due to its effect on modulation of endometrial receptivity through EMT.

## Introduction

It has been reported that around 10–15% of couples worldwide experience infertility during their reproductive years [1]. Many factors contribute to these reproductive failures, including the woman’s age, oocyte and sperm quality, parental chromosomal anomalies, and genetic or metabolic abnormalities of the embryo. Nevertheless, 75% of failed pregnancies are considered to be due to poor uterine endometrial receptivity and implantation failure [2]. Embryo implantation is a complicated physiological process that involves adhesive interaction between the trophectoderm and endometrial epithelium [3]. The endometrium is only receptive for the blastocyst to implant for a limited time span, so-called "implantation window". To be prepared for receptivity, the uterus undergoes proliferation and differentiation under the influence of ovarian hormones. In the early period of embryo implantation, the destruction of the endometrial cell barrier is prior to cell remodeling [4]. However, neither the mechanism of remodeling nor the relation between remodeling and cell migration and differentiation are clear.

Epithelial-mesenchymal transition (EMT) is the reversible biologic process that induces multiple changes in epithelial polarized cells, leading to a mesenchymal phenotype of the cells. During this process, epithelial cells lose their polarity and cell–cell adhesion, which is essential for a variety of physiological and pathological mechanisms, such as embryogenesis, differentiation, inflammation, wound healing and so on [5]. EMT allows cellular metastasis and invasion, particularly at the precancerous stage [6]. Our previous work also demonstrated that ZEB1 could induce EMT in cervical cancer metastasis [7]. It has been demonstrated that the EMT process of endometrial epithelial cells (EECs) led to cell migration, and significantly altered the remodeling of EECs with receptivity [8]. Furthermore, in vitro cell studies confirmed that EMT occurred during embryo implantation, accompanied by downregulating of E-cadherin and upregulating of vimentin expressions [9].

As a member of zinc finger protein superfamily, ZEB1 can suppress E-cadherin expression through binding to the E-BOX region of E-cadherin, so as to induce EMT and enhance the ability of cell migration and invasion [10]. ZEB1 has been demonstrated to play critical role in the development, invasion and migration of cancers [6]. Our previous work also demonstrated that ZEB1 could induce EMT in cervical cancer metastasis [7]. ZEB1 was found to be expressed in the myometrium of human uterus at the proliferative and secretory phases of menstrual cycle, and its expression could be regulated by estrogen and progesterone [11]. Our results are same as the study before, we found that poor expression of ZEB1 in the proliferative phase but a high level in secretary middle phase in human endometrium [7]. Knocking down of ZEB1 expression altered expression of E-cadherin and vimentin in EEC and inhibited the embryo implantation in cell models in vitro [7].

Owing to the expression profile of ZEB1 and its regulatory effects on the important EMT marker E-cadherin, it is likely that ZEB1 could promote EMT in EECs, but whether ZEB1 has an impact on embryo implantation has not been evaluated in vivo.

To validate the possible role of ZEB1 on EMT and embryo implantation in vivo, in the present study, the expression pattern of ZEB1 as well as EMT markers E-cadherin and vimentin, was measured in the endometrium from early-pregnant mice. Whereas shRNA technology was employed to test the effect of ZEB1 knockdown on embryo implantation in vivo.

## Materials and Methods

### Animals

Kunming mice, also known as KM mice, a specific strain of laboratory mice and an outbred strain developed in China (Specific Pathogen Free, aged 6–8 weeks with body weight 25–30 g), were obtained from the Laboratory Animal Center of Xiamen University. Mice were caged in a controlled environment with a 14 h light: 10 h dark cycle. Female and male mice were mating, according to vaginal smear, the estrus mice were chosen as nonpregnant group in this study (day 0 means nonpregnancy). Mature female mice were mated with fertile males of the same strain by caging together overnight (ratio = 2:1) and then examined in the following morning. The pregnancy (day 1) was confirmed by vaginal smears and/or the presence of a vaginal plug. Mice of pregnancy on days 1 to 7 were randomly divided into five groups (day 2.5, 3.5, 4.5, 5.5, and 6.5, n = 10 in each group). Another set of 10 pregnant mice on day 3 were used to undergo intrauterine injection for functional study of ZEB1. All animal procedures were approved by the Institutional Animal Care and Use Committee of Xiamen University, and conform to the European Convention for the Protection of Vertebrate Animals used for Experimental and other Scientific Purposes (Council of Europe No. 123, Strasbourg 1985).

### RNA Isolation and Real-Time Quantitative Reverse Transcription PCR (QRT-PCR) Analysis

RNA was isolated from mouse endometrium using RNAiso Plus (Takara, Beijing, China) following manufacturer’s protocol. Reverse transcription of total RNA to cDNA was synthesized using a Prime Script RT reagent Kit (Takara, Beijing, China) in a MyCycler Thermal Cycler (Bio-Rad, Hercules, CA, USA). Amplification reactions and thermal cycling conditions were performed by using LightCycler 480 SYBR Green I Master Mix (Roche Molecular Biochemical, Mannheim, Germany) in a LightCycler 480 System (Roche Molecular Biochemical, Mannheim, Germany) following the manufacturer’s recommendations. Primers were synthesized by Sangon Biotech (Shanghai, China), and the sequences (5'- to -3') were: Mouse ZEB1 (Zeb1, transcript variants 1–3, GenBank accession nos. NM_011546.3, NM_001360981.1 and NM_001360982.1) Forward: AAGTGGCTGTAGATGGTAACGT, Reverse: AAGGAAGACTGATGGCAGAAAT; E-cadherin (Cdh1, GenBank accession no. NM_009864.3) Forward: GACAACGCTCCCATCCCA, Reverse: CCACCTCCTTCTTCATCATAG; Vimentin (Vim, GenBank accession no. NM_011701.4) Forward: GGAAGAGAATTTTGCCCTTG, Reverse: TGGTATTCACGAAGGTGACG. Mouse β-actin (Actb, GenBank accession no. NM_007393.5) was used as reference (internal control), and primers (5'- to -3') were: Forward: CATCCGTAAAGACCTCTATGCCAAC, Reverse: ATGGAGCCACCGATCCACA. The 2-△△CT method was used to calculate the relative mRNA level of each gene.

### Western Blot

Total protein extraction from mouse endometrium, and Western blot were performed as described previously [12]. Specific primary antibodies against ZEB1 (NBP1-05987, Novus Biologicals, Wiesbaden-Nordenstadt, Germany, 1:1000), E-cadherin (ab1416, Abcam, Cambridge, UK, 1:1000), and vimentin (ab45939, Abcam, Cambridge, UK, 1:3000), GAPDH (5174, Cell Signaling Technology, Danvers, MA, USA, 1:1000), and β-actin (3700, Cell Signaling Technology, Danvers, MA, USA, 1:1000) were used.

### Immunohistochemistry

Immunohistochemical staining of mouse uteruses was performed as described previously [7], with specific antibodies against ZEB1 (1:400), E-cadherin (1:100), and vimentin (1:1000), respectively.

### Blockage of ZEB1 During Embryo Implantation In Vivo

To determine whether ZEB1 plays a role during embryonic implantation, we blocked ZEB1 function with uterine horn injection of EGFP-tagged shRNA against ZEB1 (shZEB1) or control shRNA vector (shCtrl) (Genechem, Shanghai, China) on pregnant mice. The efficiency of shRNA was verified by transfection into RL95-2 cells according to manufacturer’s instruction. The sequence of shZEB1 was: 5'-GATACACGAGCAGAACGTTA-3'. Sixteen pregnant mice on day 3 were randomly divided into two groups (8 in each group), receiving injection of shZEB1 or shCtrl to the left uterine horns, respectively, while sterile saline was injected to the right horn of each mouse. The injection volume was 5 μl. 8 days later, the mice were sacrificed by cervical dislocation. The number of embryos implanted was counted and recorded.

### Statistical Analyses

SPSS 20.0 statistical software was employed. Measurement data were expressed as mean ± standard error of mean (SEM), while t test and F test were used. The comparison of two data sets was done by t test, and more than two data sets were analyzed by using one-way analysis of variance. Homogeneity of variance was analyzed, and F test was used for multiple samples with homogeneity of variance and Welch test was used for multiple samples with no homogeneity of variance. *P* < 0.05 was considered statistically significant.

## Results

### Expression of ZEB1, E-Cadherin, and Vimentin in Mouse Endometrium of Early Pregnancy

**Fig. 1:**
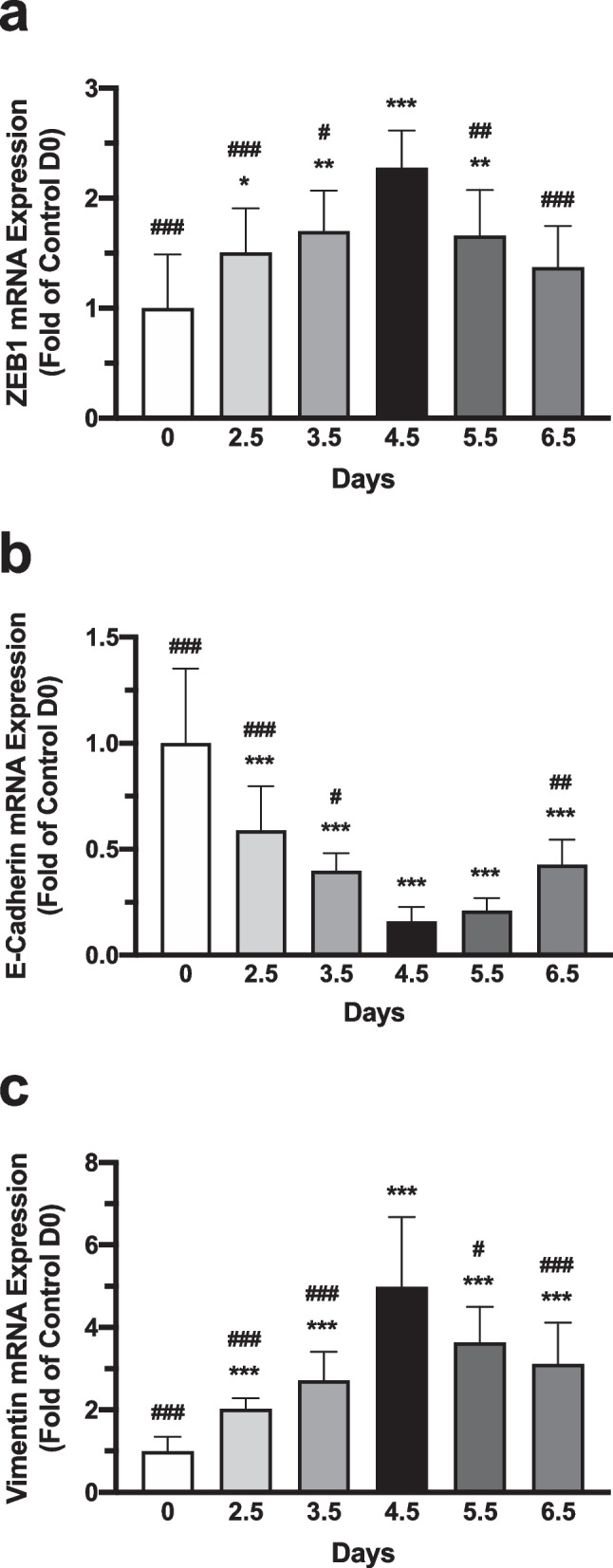
Expression of ZEB1, E-cadherin and vimentin mRNA in mouse endometria. The mRNA expression of **a** ZEB1, (**b**) E-cadherin, and (**c**) vimentin in the endometrium of pregnant and nonpregnant mice were measured by QRT-PCR. Data represent mean ± SEM, *n* = 10, *P < 0.05, **P < 0.01, ***P < 0.001 versus Ctrl (D0), ^#^*P* < 0.05, ^##^*P* < 0.01, ^###^*P* < 0.001 versus D4.5

**Fig. 2:**
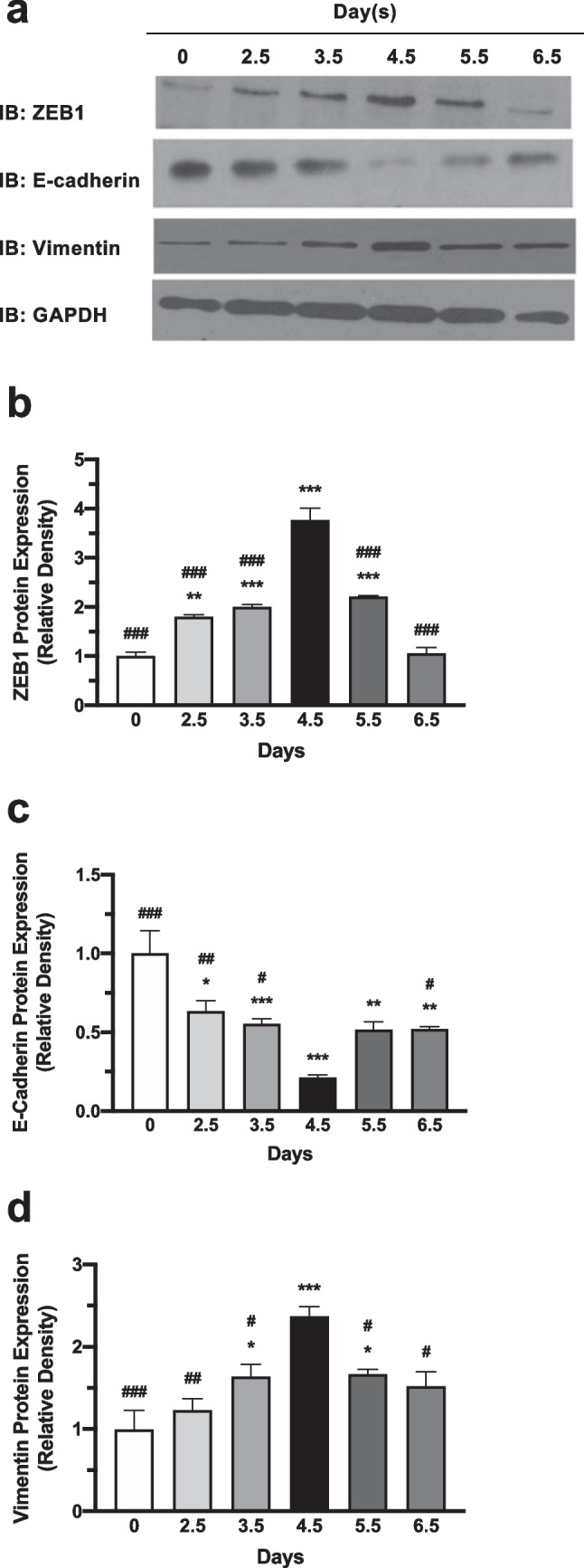
Expression of ZEB1, E-cadherin and vimentin protein in mouse endometria. (**a**) The protein expression and semi-quantitation of (**b**) ZEB1, (**c**) E-cadherin, and (**d**) vimentin in the endometrium of pregnant and nonpregnant mice were detected by Western blot. Data represent mean ± SEM, *n* = 3, *P < 0.05, **P < 0.01, ***P < 0.001 versus Ctrl (D0), ^#^*P* < 0.05, ^##^*P* < 0.01, ^###^*P* < 0.001 versus D4.5

The mRNA expression of ZEB1, and EMT makers E-cadherin and vimentin in the endometrium of pregnant and nonpregnant mice were measured by QRT-PCR (Fig. 1). High expression levels of ZEB1 and vimentin were found in pregnant mice compared to that in nonpregnant mice, regardless the days of pregnancy. It is noteworthy that the expression of ZEB1 and vimentin were markedly higher on pregnant day 4.5 than that on the other days of pregnancy (*P* < 0.001). Whereas E-cadherin mRNA expression level turned out to be lower in pregnant mice than that in nonpregnant mice, and the lowest expression was detected on pregnancy day 4.5 (*P* < 0.001), which is the implantation window for mouse. Western blot results revealed similar patterns of changes in the protein expression of ZEB1, E-cadherin and vimentin (Fig. 2). The protein expression of ZEB1 and vimentin gradually increased as pregnant days passed, reached a high peak on day 4.5 (*P* < 0.001), and started to decline from day 5.5. On the contrary, the protein expression of E-cadherin decreased from day 0 to day 4.5 (*P* < 0.001), and recovered thereafter.

### Immunohistochemical Staining of Mouse Uterus of Early Pregnancy

**Fig. 3:**
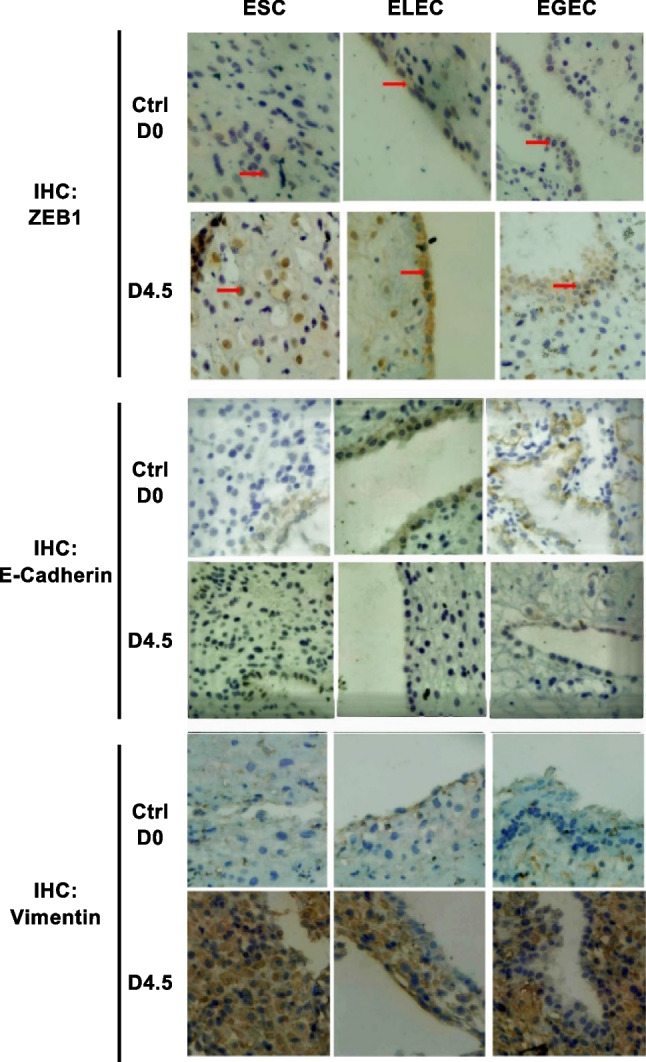
Immunohistochemistry staining of ZEB1, E-cadherin and vimentin in mouse endometrium. The results showed positive expression of ZEB1 in the endometrium of pregnant, and faint signals were detected in nonpregnant mice. ZEB1 was located mainly in early pregnant luminal and stromal cells, partly located in glandular epithelium. The strong immunoreactivity for ZEB1 was observed in mouse epithelium on pregnant day 4.5. Similar expression pattern and localization were detected for vimentin. Weak positive staining of vimentin was found in the endometrium of nonpregnant mice and strong immunoreactivity for vimentin was observed in stromal cells, and luminal and glandular epithelium on pregnant day 4.5. E-cadherin protein was decreased in luminal and glandular epithelium as the days passed in early stage of pregnancy, Lowest E-cadherin protein was detected in the epithelium and stroma cells on pregnant day 4.5

Immunohistochemical staining showed positive expression of ZEB1 in the luminal and gland epithelium and stromal cells of in the endometrium of pregnant mice (Fig. 3). Only faint signals were detected in nonpregnant mice. ZEB1 was located mainly in early pregnant luminal and stromal cells, partly located in glandular epithelium. The strong immunoreactivity for ZEB1 was observed in mouse epithelium on pregnant day 4.5. Similarly, weak positive staining of vimentin was found in the endometrium of nonpregnant mice, and strong immunoreactivity for vimentin was observed in stromal cells, and luminal and glandular epithelium on pregnant day 4.5. However, for a higher rate of positive distribution was seen in both luminal and glandular epithelia of nonpregnant mice, and not in stromal cells. During pregnancy, E-cadherin protein was decreased in luminal and glandular epithelium as the days passed in early stage of pregnancy. Lowest E-cadherin protein was detected in the epithelium and stroma cells on pregnant day 4.5.

### Blockage of ZEB1 Suppressed Embryo Implantation in Mice

**Fig. 4:**
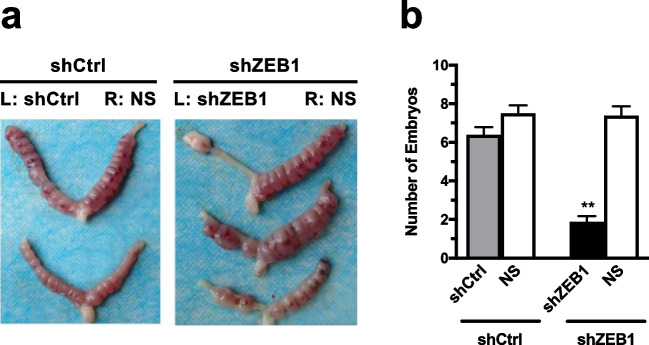
ZEB1 knockdown suppressed embryo implantation in mice in vivo. (**a**) The number of embryos implanted after intrauterine injection with shZEB1 was compared with control group (shCtrl). (**b**) The embryo implantation rate calculated. Data represent mean ± SEM, *n* = 8, **P < 0.01

To confirm the effects of ZEB1 on embryo implantation in vivo, the number of embryos implanted after intrauterine injection with shZEB1 was compared with control group (shCtrl). As shown in Fig. 4, the total number of embryos implanted in shZEB1 group was less than that in shCtrl group. The embryo implantation rate in shZEB1 group was significantly lower than the saline side of the uterus (1.875 ± 0.835 versus 7.375 ± 1.408, t = 9.505, *P* < 0.01). Whereas the embryo implantation rate in shCtrl group did not differ from the saline side of the uterine (6.375 ± 1.119 versus 7.500 ± 1.119, t = 1.888, *P* > 0.05).

## Discussion

Successful embryo implantation requires the synchronous development of the receptive uterus and competent blastocyst [13]. Although in vitro fertilization and embryo transfer techniques have been partially successful in overcoming human infertility, the embryo implantation rates remain disappointingly low, which may be partially due to the presence of a nonreceptive uterus at the time of embryo implantation [14]. During implantation, the uterus undergoes proliferation and differentiation under the influence of ovarian hormones in order to acquire receptivity [15]. It was reported that the differentiation of endometrial epithelial cells and stromal cells is regulated by steroid hormones during menstrual cycle so as to ensure that the embryonic receptivity of endometrium occurs on 7 to 11 days after ovulation, which normally occurs on days 4 to 5 of pregnancy in mice [16]. This is a precise and complex process that requires the participation of various molecules. However, in the event that the genes associated with the establishment of uterine receptivity are expressed abnormally, embryo implantation may be affected directly, and then spontaneous abortion occurs [17]. The remodeling of epithelium barrier is a key process for uterine receptivity, during which EMT may play a important role [8].

EMT is essential for a variety of physiological and pathological process [5]. It is well documented that EMT is one of the important mechanisms of carcinoma metastasis [18]. Among the cluster of genes that regulate EMT process, ZEB1 is considered to be a key transcription factor [19]. Aberrant expression of ZEB1 in a subpopulation of tumor cells is a marker of biologically more aggressive uterine malignancies [20]. In lung and mammary gland tumor cells, ZEB1 expression was altered to promote EMT, which is associated with tumor metastasis [21], and was aberrantly expressed in epithelial cells of aggressive endometrial cancers [22]. However, the role of ZEB1 in embryo implantation has not been clearly demonstrated in vivo.

We here examined the expression pattern of ZEB1 in EECs from mouse uterus during early pregnancy. Higher expression of ZEB1 mRNA and protein was detected in the pregnant group compared to that in the nonpregnant group, presenting an upward trend from day 2.5 to 4.5, and reached the peak on day 4.5 of pregnancy. Day 4.5 is considered to be the implantation window of pregnant mice [16]. ZEB1 is concentrated at the mouse endometrium of “implantation window”, which similary to the human [7], so we speculate that ZEB1 play an important role in embryo implantation. It is Immunohistochemical staining results showed that ZEB1 located mainly in early pregnant luminal cells and stromal cells nearby, and partly located in glandular epithelium. The strong immunoreactivity for ZEB1 was observed in mouse epithelium on pregnant day 4.5, and only faint signal was detected in nonpregnant mice endometrium. Additionally, ZEB1 expression was higher in luminal cells than it was in glandular epithelium, suggesting that ZEB1 was mainly involved in the EEC barrier disruption via EMT, in order to provide an area for the embryo expansion. ZEB1 expressed in stromal cells simultaneously, indicating that ZEB1 might be involved in the remodeling of the disrupted EEC barrier as well.

"Epithelial phenotypes" was classified by high E-cadherin and low vimentin expressions. Conversely, "mesenchymal phenotypes" was classified by low E-cadherin and high vimentin expressions [23]. Besides high expression of ZEB1, we found decreased E-cadherin and increased vimentin expressions in mouse endometrium on day 4.5 of pregnancy. Vimentin is the major constituent of intermediate filaments in normal and neoplastic mesenchymal cells, and is normally expressed in the mesenchymal tissue [24]. Growing evidence suggested that the aberrant expression of vimentin in epithelial cancer cells facilitates the acquisition of invasive and metastatic properties [25]. It is intriguing that vimentin showed similar expression pattern to ZEB1 in early pregnant mice. It was poorly expressed on pregnant day 0, but was increased with the pregnant day passed, and reached the peak on day 4.5. This indicated that increased ZEB1 expression in mouse uterine of "implantation window" might up-regulate vimentin to promote EMT, facilitating the migration of EECs away from the implantation site. Concurrently, down-regulation of E-cadherin was observed in pregnant mice endometrium. The expression of E-cadherin mRNA and protein was at the highest level on day 0 of pregnancy, and markedly declined on day 4.5 to 5.5. Positive expression of E-cadherin is mainly located in luminal and glandular cells. E-cadherin is an adhesion molecule and plays an important role in the adhesion between epithelial cells. The loss of E-cadherin in EECs on day 4.5 to 5.5 suggested that the EEC barrier is disrupted via EMT. Therefore, during the implantation of mice, EMT is likely to be trigged by overexpression of ZEB1 through the altered expression of E-cadherin and vimentin.

To further confirm the role of ZEB1 on the regulation of mouse implantaiton, we employed shRNA technology to suppress ZEB1 in vivo. Administration of shZEB1 to uterine horn significantly reduced the number of embryos implanted in the pregnant mice, compared to that of shCTRL group. We previously reported that knockdown of ZEB1 expression in cervical cell line Caski cells resulted in attenuated proliferation and migration abilities, paralleled with altered expression of EMT-related genes E-cadherin and vimentin [7]. In combination of the data in the present study, the importance of ZEB1 in endometrial receptivity and embryo implantation is further confirmed in vivo. This could at least partially explain the cause of implantation failure associated with endometrial receptivity.

In conclusion, ZEB1 plays a vital role in the process of embryo implantation of mice. It is likely that ZEB1 regulates the process of embryo implantation via alteration of E-cadherin and vimentin expression and promotion of EMT in pregnant mice. These findings might be beneficial for a better understanding of the role of ZEB1 during embryo implantation. Further studies particularly on ZEB1-related mechanisms are required to expand the in-depth knowledge about infertility.

## Data Availability

The authors confirm the availability of data and materials, ensure data transparency, and confirm the availability of the code involved.

## References

[1] Schulte MM, Tsai JH, Moley KH. Obesity and PCOS: the effect of metabolic derangements on endometrial receptivity at the time of implantation. Reprod Sci. 2015;22(1):6–14. 25488942 10.1177/1933719114561552 PMC4275454

[2] Zipori Y, et al. Multifetal pregnancy reduction of triplets to twins compared with non-reduced triplets: a meta-analysis. Reprod Biomed Online. 2017;35(3):296–304. 28625760 10.1016/j.rbmo.2017.05.012

[3] Lim W, et al. Characterization of C-C motif chemokine ligand 4 in the porcine endometrium during the presence of the maternal-fetal interface. Dev Biol. 2018;441(1):146–58. 30056935 10.1016/j.ydbio.2018.06.022

[4] Matson BC, et al. Adrenomedullin improves fertility and promotes pinopodes and cell junctions in the peri-implantation endometrium. Biol Reprod. 2017;97(3):466–77. 29025060 10.1093/biolre/iox101 PMC6248476

[5] Yamada N, et al. Tumor budding at the invasive front of colorectal cancer may not be associated with the epithelial-mesenchymal transition. Hum Pathol. 2017;60:151–9. 27836787 10.1016/j.humpath.2016.10.007

[6] Orellana-Serradell O, et al. The transcription factor ZEB1 promotes an aggressive phenotype in prostate cancer cell lines. Asian J Androl. 2018;20(3):294–9. 29271397 10.4103/aja.aja_61_17 PMC5952486

[7] Ran J, et al. ZEB1 promotes epithelial-mesenchymal transition in cervical cancer metastasis. Fertil Steril. 2015;103(6):1606-14e1-2. 25963537 10.1016/j.fertnstert.2015.03.016

[8] Du F, et al. Expression of transcriptional repressor Slug gene in mouse endometrium and its effect during embryo implantation. Appl Biochem Biotechnol. 2009;157(2):346–55. 19172233 10.1007/s12010-008-8521-8

[9] Uchida H, et al. Studies using an in vitro model show evidence of involvement of epithelial-mesenchymal transition of human endometrial epithelial cells in human embryo implantation. J Biol Chem. 2012;287(7):4441–50. 22174415 10.1074/jbc.M111.286138 PMC3281640

[10] Maffeis V, et al. Tumor budding is an adverse prognostic marker in intestinal-type sinonasal adenocarcinoma and seems to be unrelated to epithelial-mesenchymal transition. Virchows Arch. 2020;477(2):421–248. 31980958 10.1007/s00428-020-02748-1

[11] Wu RF, et al. Lipoxin A4 suppresses estrogen-induced epithelial-mesenchymal transition via ALXR-dependent manner in endometriosis. Reprod Sci. 2018;25(4):566–78. 28691579 10.1177/1933719117718271

[12] Wu R, et al. Lipoxin A4 suppresses the development of endometriosis in an ALX receptor-dependent manner via the p38 MAPK pathway. Br J Pharmacol. 2014;171(21):4927–40. 24923883 10.1111/bph.12816 PMC4294115

[13] Matsumoto H. Molecular and cellular events during blastocyst implantation in the receptive uterus: clues from mouse models. J Reprod Dev. 2017;63(5):445–54. 28638003 10.1262/jrd.2017-047 PMC5649093

[14] Elsokkary M, et al. The reproducibility of the novel utilization of five-dimensional ultrasound and power Doppler in the prediction of endometrial receptivity in intracytoplasmic sperm-injected women: a pilot prospective clinical study. Arch Gynecol Obstet. 2019;299(2):551–8. 30564930 10.1007/s00404-018-5001-4

[15] Long X, et al. Expression of KRAS in the endometrium of early pregnant mice and its effect during embryo implantation. Reprod Biomed Online. 2015;31(1):51–61. 25999213 10.1016/j.rbmo.2015.04.005

[16] Fullerton PT Jr, et al. Follistatin is critical for mouse uterine receptivity and decidualization. Proc Natl Acad Sci U S A. 2017;114(24):E4772–81. 28559342 10.1073/pnas.1620903114 PMC5474784

[17] Patel JA, et al. Personalized embryo transfer helps in improving in vitro fertilization/ICSI outcomes in patients with recurrent implantation failure. J Hum Reprod Sci. 2019;12(1):59–66. 31007469 10.4103/jhrs.JHRS_74_18 PMC6472200

[18] Chen J, et al. The aberrant expressions of MACC1, ZEB1, and KLF4 in hepatocellular carcinoma and their clinical significance. Int J Clin Exp Pathol. 2019;12(9):3653–61. 31934216 PMC6949830

[19] Moussa RA, Khalil EZI, Ali AI. Prognostic role of epithelial-mesenchymal transition markers “E-cadherin, beta-catenin, ZEB1, ZEB2 and p63” in bladder carcinoma. World J Oncol. 2019;10(6):199–217. 31921376 10.14740/wjon1234 PMC6940035

[20] Xu J, et al. The inhibition of miR-126 in cell migration and invasion of cervical cancer through regulating ZEB1. Hereditas. 2019;156:11. 31007650 10.1186/s41065-019-0087-7 PMC6456986

[21] Bakiri L, et al. Fra-1/AP-1 induces EMT in mammary epithelial cells by modulating Zeb1/2 and TGFbeta expression. Cell Death Differ. 2015;22(2):336–50. 25301070 10.1038/cdd.2014.157 PMC4291495

[22] Spoelstra NS, et al. The transcription factor ZEB1 is aberrantly expressed in aggressive uterine cancers. Cancer Res. 2006;66(7):3893–902. 16585218 10.1158/0008-5472.CAN-05-2881

[23] Kalluri R, Weinberg RA. The basics of epithelial-mesenchymal transition. J Clin Invest. 2009;119(6):1420–8. 19487818 10.1172/JCI39104 PMC2689101

[24] Li Y, et al. Effects of UPF1 expression on EMT process by targeting Ecadherin, Ncadherin, Vimentin and Twist in a hepatocellular carcinoma cell line. Mol Med Rep. 2019;19(3):2137–43. 30628676 10.3892/mmr.2019.9838 PMC6390072

[25] Satelli A, et al. EMT circulating tumor cells detected by cell-surface vimentin are associated with prostate cancer progression. Oncotarget. 2017;8(30):49329–37. 28521303 10.18632/oncotarget.17632 PMC5564771

